# Associations between Phase Angle Values Obtained by Bioelectrical Impedance Analysis and Nonalcoholic Fatty Liver Disease in an Overweight Population

**DOI:** 10.1155/2020/8888405

**Published:** 2020-08-04

**Authors:** Guangya Chen, Yi Lv, Wei Ni, Qingxin Shi, Xingliang Xiang, Sen Li, Chengwu Song, Mingzhong Xiao, Shuna Jin

**Affiliations:** ^1^College of Pharmacy, Hubei University of Chinese Medicine, Wuhan, Hubei, China; ^2^Hepatic Disease Institute, Hubei Provincial Hospital of Traditional Chinese Medicine, Wuhan, Hubei, China; ^3^Hubei Provincial Academy of Traditional Chinese Medicine, Wuhan, Hubei, China; ^4^Department of Pharmacy, Union Hospital, Tongji Medical College, Huazhong University of Science and Technology, Wuhan, Hubei, China; ^5^Key Laboratory of Environment and Health, Ministry of Education and Ministry of Environmental Protection, State Key Laboratory of Environmental Health, School of Public Health, Tongji Medical College, Huazhong University of Science and Technology, Wuhan, Hubei, China

## Abstract

**Objective:**

There is a limited diagnosis of nonalcoholic fatty liver disease (NAFLD). Thus, the noninvasive assessments are worth exploring. We determined the associations of phase angles (PhAs) obtained from bioelectric impedance analysis (BIA) with the risk of NAFLD in an overweight population.

**Methods:**

A study involving 953 overweight participants was conducted in Wuhan city, China. The associations between PhAs (right arm, left arm, body trunk, right leg, left leg, and whole body) and the risk of NAFLD were conducted using multivariate logistic regression analyses. The associations of PhAs with the controlled attenuation parameter (CAP), a noninvasive assessment of liver steatosis and fibrosis, were also evaluated by both linear and logistic regression analyses.

**Results:**

The PhA values of the whole body, trunk, and legs were significantly lower (*P* < 0.05) in the NAFLD group than the non-NAFLD group. After adjustment for BMI, gender, education, income/year, hyperlipidemia, hypertension, diabetes, smoking, passive smoking, and drinking, significant associations of PhA values of the right leg, left leg, and whole body with the risk of NAFLD were observed. In addition, the PhA of the right leg, left leg, and whole body were significantly related to the CAP values. Further stratified analyses indicated that these associations were significant in the participants with BMI <30, but not in the participants with BMI ≥30.

**Conclusions:**

PhAs might be effective indicators in the management of NAFLD among overweight people.

## 1. Introduction

Nonalcoholic fatty liver disease (NAFLD) is a clinicopathological liver disease, which is characterized by macrovesicular hepatic lipids accumulation and the occurrence in patients who consume little or even no alcohol. NAFLD is not a single disease and usually can be divided into three stages: nonalcoholic fatty liver, nonalcoholic steatohepatitis, and liver cirrhosis [1]. With the increase of the obesity epidemic throughout the world, NAFLD is one of the most common liver diseases both in the developed and developing world with an estimated prevalence of 20–40% [2]. Although the exact prevalence varies from region to region, the overall trend shows an increased number of patients with NAFLD. In the North of the Netherlands, 22% of adults suffered from NAFLD [3]. A recent report in Beijing indicated that the prevalence of NAFLD in adults was 31.0% in the Asiatic population [4]. NAFLD has rapidly become a leading indication of liver transplantation because of its increasing prevalence and lack of effective therapies [5].

Until now, the pathogenesis of NAFLD is not fully clear. High-fat diets and physical inactivity are strongly associated with the development and progression of NAFLD [6]. In addition, there was evidence that increased body fat, particularly abdominal visceral fat, was central in the pathogenesis of NAFLD [7, 8]. However, it is a fact that not all overweight people have NAFLD. Therefore, it is critical to explore early diagnosis methods or indicators to prevent the occurrence of NAFLD in the high incidence population, such as overweight or obese people.

The diagnosis of NAFLD requires imaging or histological evidence of an excessive accumulation of triglyceride in hepatocytes. The gold standard for the diagnosis remains the percutaneous liver biopsy. However, because of increased cost, higher risk, and health care resource use, invasive liver biopsy is a poorly suited diagnostic test for such a prevalent disease [9]. Bioelectrical impedance analysis (BIA) is a noninvasive tool for assessing body composition [10]. It measures the resistance and capacitance of body components by recording a voltage drop in the applied current [11]. Capacitance causes the current to lag behind the voltage, resulting in a phase shift. This shift is quantified geometrically as the angular transformation—the phase angle (PhA). Previous reports showed that the PhA was strongly associated with a poor outcome in clinical conditions. Gupta et al. demonstrated that PhA was a strong predictor of survival in breast cancer [12]. Two cross-sectional studies in Germany indicated that higher PhA was associated with lower relative mortality risk [13]. A case-control study including 983 healthy adults and 983 admitted patients suggested a significant association between low PhA and nutritional risk [14]. However, it remains unclear whether PhA can be a predictor for the risk of NAFLD in overweight people.

Therefore, we sought to determine the associations of NAFLD with PhAs of the whole body, trunk, arms, and legs obtained from BIA in an overweight population. In addition, the associations between PhAs and the controlled attenuation parameter (CAP), a noninvasive assessment of liver steatosis and fibrosis, were also investigated.

## 2. Materials and Methods

### 2.1. Study Subject

This study recruited 953 overweight participants at Hubei Provincial Hospital of Traditional Chinese Medicine in 2016. The overweight was defined as a body mass index (BMI) ≥ 24.0 kg/m^2^ according to the Chinese reference standard. The participants met the following criteria: (1) BMI ≥ 24.0 kg/m^2^, (2) aged 18–60, (3) written informed consent, and (4) ability to comprehend the Chinese language and complete the questionnaire. Besides, all participants provided written informed consent at enrollment, and the ethics protocol was acquired by the Ethics Committee of Hubei Provincial Hospital of Traditional Chinese Medicine.

### 2.2. Data Collection

We used structured and validated questionnaires to obtain information on socioeconomic and lifestyle characteristics, such as age, education, income/year, frequency of alcohol intake (how many times per week), and smoking status. Height and body weight were measured in participants wearing light clothing without shoes. BMI was calculated as body weight in kilograms divided by the square of height (kg/m^2^). Hypertension was defined as Diastolic Blood Pressure ≥90 mmHg and (or) Systolic Blood Pressure ≥ 140 mmHg at examinations or having hypertension history. Diabetes was defined as fasting plasma glucose ≥7.0 mmol/L or having diabetes history.

### 2.3. PhA Determination

PhA was assessed by a bioelectric impedance device (InBdoy 770, InBody, China). The test procedure was conducted according to the manufacturer's instructions. Participants removed all metal objects and other items that might interfere with the scan and emptied the bladder. Participants stepped on the multifrequency BIA device with bare feet, held hands on the hand rails, and stayed on the device for two minutes. The analyzer uses an alternate current of 80 mA and assesses the PhA at 50 kHz. The MF-BIA device measures segmental impedances at the right arm, left arm, right leg, left leg, and trunk for six frequencies. The PhA for each half of the body at each frequency is then calculated using the following formula: PhA = arctan(Xc/*R*) × 180/*π* (Xc is the reactance and *R* is the body resistance).

### 2.4. NAFLD Diagnosis and CAP Determination

The diagnosis of NAFLD in this study was determined by abdominal echography with the characteristic of “bright liver” and evidence of hepatic and renal parenchyma, vessel blurring, focal sparing, and hepatic veins stenosis, according to the Chinese Guidelines for the Diagnosis and Management of Nonalcoholic Fatty Liver Disease (update in 2010) [15]. Ultrasonography was performed by experienced radiologists to diagnose NAFLD.

Currently, because CAP has been reported to perform well for mild steatosis on people with NAFLD [16], the normal range was less than 240 dB/min and our study also tested CAP values. CAP was measured by the ultrasonic attenuation at 3.5 MHz using the FibroScan (EchoSens, Paris, France). CAP was developed on the postulate that fat affected ultrasonic propagation. The value was based on the degree of ultrasonic attenuation by hepatic fat at the central frequency of the FibroScan® M probe simultaneously with liver stiffness measurement [17].

### 2.5. Statistical Analysis

All statistical analyses were performed using SAS (version 9.4; SAS Institute Inc.). Data were expressed in means with standard deviations (SD) and odds ratio (OR) with a 95% confidence interval (CI). The PhA values at a frequency of 50 kHz in NAFLD and control people were compared by independent sample *t*-tests. The associations between NAFLD and PhAs (right arm; left arm; body trunk; right leg; left leg; whole body) were estimated using logistic regression models. The relationships between CAP and PhAs (right arm; left arm; body trunk; right leg; left leg; whole body) were explored using linear regression models. Logistic regression analyses were also used to estimate the associations between CAP and PhAs with a cut-off at 240 dB/min for CAP. Besides, we also conducted stratified analyses by BMI (BMI <30 and BMI ≥30) to explore the differences in the associations of PhA with NAFLD and CAP among different BMI groups. Covariates were selected as potential confounders because they were reported to be associated with NAFLD or the *P*-value <0.1 on the basis of the statistical consideration. Age, BMI, gender, hyperlipidemia hypertension, diabetes, smoking, passive smoking, drinking, education, and income were adjusted in the final models [18–21]. Two-sided *P*-values of <0.05 were considered statistically significant.

## 3. Results

Table 1 lists the characteristics of 953 participants with BIA assessment. There were 628 female and 325 male participants in our study. The mean age was 44.0 ± 9.7 years. The BMI in 24.0–26.9 kg/m^2^ was 39.0%, in 27.0–29.9 kg/m^2^ was 36.2%. The participants of less than high school were 30.6% and more than high school were 30.5%. About 33.3% of people had a family income of less than 50000 yuan per year. There were 15.6% of participants with drinking, 19.6% with smoking, and 64.6% with passive smoking. In our study population, 12.9%, 19.10%, and 5.35% of participants had suffered from hyperlipidemia, hypertension, and diabetes, respectively. In addition, there were 271 non-NAFLD and 682 NAFLD participants in our population.

The levels of PhA in different body parts and CAP are shown in Table 2. The results indicated that the trunk PhA had the highest mean level and trunk PhA levels in the NAFLD group were significantly higher than the non-NAFLD group (*P* = 0.039). Besides, PhA levels of the right leg, left leg, and whole body in the NAFLD group were also higher than the non-NAFLD group (all *P* < 0.05). However, the PhA levels of both the right and left arms had no differences between the NAFLD and non-NAFLD groups. Meanwhile, the CAP levels in the NAFLD group were differed by levels in the non-NAFLD group and the participants with NAFLD disease had higher CAP levels (*P* < 0.001).

Table 3 displays the relationships of NAFLD with PhAs and the characteristics of study participants. We found that participants' age, BMI, hyperlipidemia, hypertension, and diabetes were associated with NAFLD in univariate analysis (all *P* < 0.01). When the age, BMI, gender, education, income/year, hyperlipidemia, hypertension, diabetes, smoking, passive smoking, drinking, and the PhA were included in the multivariate model, there were still significant associations of NAFLD with the participants' age, BMI, and hyperlipidemia. Besides, the relationships of PhAs in six body parts (right arm, left arm, trunk, right leg, left leg, and whole body) with the risk of NAFLD were further investigated. Our results showed that PhAs of the trunk, right leg, left leg, and whole body were associated with the risk of NAFLD in univariate analysis (all *P* < 0.05). When in multivariate models, significant associations were found in the PhAs of the right leg, left leg and whole body (for right leg, OR = 1.41, 95% CI: 1.08 to 1.83; for left leg, OR = 1.30, 95% CI: 0.01 to 1.66; for whole body, 1.40, 95% CI: 1.03 to 1.91; all *P* < 0.05).

The associations between CAP and PhAs at frequency of 50 kHz are shown in Table 4. We conducted the univariate and multivariate linear regression between CAP and PhAs of six body parts. In multivariate analyses, the PhAs of the right leg, left leg, and whole body showed significantly linear associations with CAP values (for right leg, *β* = 9.69, 95% CI: 4.79 to 14.59; for right leg, *β* = 9.40, 95% CI: 4.33 to 14.46; for whole body, *β* = 7.20, 95% CI: 1.32 to 13.09; all *P* < 0.05). Besides, logistic regression analyses were also used to estimate the associations between CAP and PhAs with a cut-off at 240 dB/min for CAP. The results suggested that CAP were also significantly associated with the PhAs of the right leg, left leg and whole body (for right leg, OR = 1.45, 95% CI: 1.15 to 1.82; for left leg, OR = 1.43, 95% CI: 1.13 to 1.81; for whole body, OR = 1.34, 95% CI: 1.02 to 1.76; all *P* < 0.05).

The stratified analyses by BMI are shown in Table 5. In the multivariate logistic regression models, there was no PhA associated with NAFLD and abnormal CAP in the participants with BMI ≥30. While in the participants with BMI <30, the associations of the PhAs of the right leg, left leg, and whole body with NAFLD were significant (for the right leg, OR = 1.70, 95% CI: 1.30 to 2.22; for the left leg, OR = 1.53, 95% CI: 1.17 to 2.00; for the whole body, OR = 1.65, 95% CI: 1.20 to 2.28; all *P* < 0.05). Besides, the PhAs of the right leg, left leg, and whole body were also associated with abnormal CAP (for the right leg, OR = 1.58, 95% CI: 1.22 to 2.05; for the left leg, OR = 1.49, 95% CI: 1.15 to 1.93; for the whole body, OR = 1.47, 95% CI: 1.08 to 2.00; all *P* < 0.05).

## 4. Discussion

In the present study, our findings showed that PhAs were significantly associated with the risk of NAFLD. The associations were still robust after further adjustment for potential confounders. Besides, we found significant associations between PhAs and CAP values, a common test for clinical diagnosis of NAFLD. The results suggested that PhA values might be effective indicators in the management of NAFLD among overweight people.

BIA has a lot of advantages, including noninvasive, maneuverable, objectivity, reproducibility, and low cost. BIA device can measure various body compositions, such as Lean Body Mass, body cell mass, Extracellular water, intracellular water, fat mass, fat free mass, and so on. In contrast to BIA-derived body composition, i.e., fat mass and fat free mass, PhA does not depend on equations and their inherent assumptions [22]. BIA measures PhA through to bioelectricity parameters: body resistance (*R*) and reactance (Xc). PhA can be expressed as arctan Xc/*R*. Resistance, the restriction of the flow of electrical current, is primarily related to the water in the tissues; reactance, the resistive effect produced by the tissue interfaces and cell membranes, can represent the cell membrane integrity [23]. Accumulating evidence has proposed that PhA could be a useful indicator for several clinical states, including cancer, cirrhosis, sarcopenia chronic obstructive pulmonary disease, and acute respiratory failure [24–27].

There was evidence that higher BMI was associated with increased PhA values because a higher BMI person tended to have more cells (fat or muscle cells) [28, 29]. Lower PhA indicated decreased cell integrity and might be an accurate predictor of poor prognosis [11, 30]. Previous studies had confirmed that hepatocellular steatosis and excessive accumulation of extra-and intrahepatic fats was the hallmark of NAFLD. A study including 75 healthy adults and 48 NAFLD patients suggested that total body fat mass was significantly associated with the risk of NAFLD [31]. Our results were consistent with these reports. The findings that PhAs of the right leg, left leg, and whole body were significantly associated with the risk of NAFLD and CAP supports PhAs can be indicators to the prevention and management of NAFLD. However, the associations of the PhAs with NAFLD and abnormal CAP were only observed in the participants with BMI <30, but not in the participants with BMI ≥30. In our study, the percentage of NAFLD was very high in the people with BMI ≥30, at about 93%. Thus no significant associations were found in this group.

Our results indicated that significant associations were found in the PhAs of the right leg, left leg, and whole body, while the associations were not found in arms and truck PhA values. In the arm measurement with BIA, the high estimation error may occur due to the small tissue interface and cell number in arms. A previous report compared BIA with dual-energy X-ray absorptiometry in young wrestlers, showing that there was no high correlation of body composition in the arm [32]. As for the trunk, PhA values of trunk showed the lowest correlation with other parts in our study (Table S1). The liquid- and air-filled spaces of trunk can have an impact on the resistivity. Compared with limbs, the trunk has a much larger cross-sectional area, which may also cause its surface resistance to be very small. Nevertheless, muscle fibers of the truck are multidirectional and the current needs to pass through many different tissue interfaces, resulting in an increase of trunk resistance [33]. Thus, the trunk PhA is affected by these factors in the measurement.

To our knowledge, this is the first study attempting to establish a relationship between the PhA and NAFLD in an overweight population. In this study, we found that higher PhA was significantly associated with the risk of NAFLD. After controlling for confounders, the associations remained significant. There are some limitations to this study that should also be taken into account. First, the data were collected at a single care center. Then, it was unclear if we could extrapolate our findings to a normal weight population because our participants were all overweight. Further research is necessary to investigate the association between PhA and NAFLD in a normal weight population.

## 5. Conclusion

Our study has indicated that PhAs were significantly associated with the risk of NAFLD in an overweight population. Positive associations between PhAs and CAP values were also observed. The findings provide evidence that PhAs might be effective indicators in the prognosis of NAFLD in overweight people. Further investigations are needed to explore PhA in a larger population.

## Figures and Tables

**Table 1:** Baseline characteristics (*N* = 953).

Characteristics	Mean ± SD or *N* (%)
Age (years)	44.0 ± 9.7
≤40	325 (34.1%)
40–51	318 (33.4%)
>51	310 (32.5%)
BMI (kg/m^2^)	28.4 ± 3.1
24.0–26.9	372 (39.0%)
27.0–29.9	345 (36.0%)
≥30	235 (24.7%)
Missing	1 (0.1%)
Gender	
Male	325 (34.1%)
Female	628 (65.9%)
Education	
Less than high school	292 (30.6%)
High school	370 (38.8%)
More than high school	291 (30.5%)
Income/year (yuan)	
<50000	317 (33.3%)
50000–100000	453 (47.5%)
>100000	182 (19.1%)
Missing	1 (0.1%)
Fatty liver	
Yes	271 (28.4%)
No	682 (71.6%)
Hyperlipidemia	
Yes	123 (12.9%)
No	830 (87.1%)
Hypertension	
Yes	182 (19.1%)
No	771 (80.9%)
Diabetes	
Yes	51 (5.4%)
No	902 (94.6%)
Smoking	
Yes	187 (19.6%)
No	765 (80.3%)
Missing	1 (0.1%)
Passive smoking	
Yes	616 (64.6%)
No	332 (34.8%)
Missing	5 (0.5%)
Drinking	
Yes	149 (15.6%)
No	804 (84.4%)

**Table 2:** Phase angles at a frequency of 50 kHz in the study participants.

	Total (*N* = 953)	Non-NAFLD (*N* = 271)	NAFLD (*N* = 682)	*P* value
Right arm	5.10 ± 0.64	5.07 ± 0.60	5.11 ± 0.66	0.36
Left arm	4.94 ± 0.64	4.90 ± 0.60	4.96 ± 0.66	0.18
Trunk	7.20 ± 1.14	7.09 ± 1.08	7.26 ± 1.12	**0.04**
Right leg	5.86 ± 0.75	5.76 ± 0.73	5.90 ± 0.76	**0.01**
Left leg	5.81 ± 0.74	5.72 ± 0.71	5.84 ± 0.75	**0.03**
Whole body	5.50 ± 0.65	5.43 ± 0.60	5.53 ± 0.66	**0.04**
CAP	251.58 ± 47.86	221.98 ± 45.74	263.24 ± 43.48	**<0.001**

All *P* values were calculated using independent sample *t*-tests. CAP: controlled attenuation parameter. *P* values of < 0.05 were considered statistically significant.

**Table 3:** Univariate and multivariate logistic regression analyses between phase angles and NAFLD.

Characteristics	Univariate analysis		Multivariate analyses^a^	
	OR (95% CI)	*P* value	OR (95% CI)	*P* value
Age	1.02 (1.01 to 1.03)	**<0.01**	1.03 (1.01 to 1.05)	**<0.01**
BMI	3.36 (2.68 to 4.22)	**<0.01**	3.21 (2.54 to 4.06)	**<0.01**
Gender	0.82 (0.61 to 1.11)	0.20	0.96 (0.60 to 1.53)	0.85
Education	0.97 (0.81 to 1.16)	0.76	1.19 (0.94 to 1.50)	0.14
Income/year	1.03 (0.84 to 1.25)	0.79	1.01 (0.80 to 1.27)	0.95
Hyperlipidemia	4.20 (2.28 to 7.76)	**<0.01**	3.08 (1.59 to 5.97)	**<0.01**
Hypertension	2.20 (1.45 to 3.34)	**<0.01**	1.26 (0.78 to 2.03)	0.34
Diabetes	3.84 (1.51 to 9.78)	**<0.01**	2.05 (0.75 to 5.58)	0.16
Smoking	1.23 (0.85 to 1.77)	0.28	1.14 (0.70 to 1.86)	0.60
Passive smoking	1.00 (0.75 to 1.35)	0.99	0.94 (0.68 to 1.31)	0.73
Drinking	0.81 (0.55 to 1.18)	0.27	0.71 (0.44 to 1.13)	0.15
*Phase angles*				
Right arm	1.11 (0.89 to 1.39)	0.36	1.15 (0.84 to 1.56)	0.38
Left arm	1.16 (0.93 to 1.45)	0.18	1.22 (0.90 to 1.65)	0.21
Trunk	1.15 (1.01 to 1.31)	**0.04**	1.10 (0.94 to 1.28)	0.25
Right leg	1.27 (1.05 to 1.54)	**0.01**	1.41 (1.08 to 1.83)	**0.01**
Left leg	1.24 (1.03 to 1.51)	**0.03**	1.30 (1.01 to 1.66)	**0.05**
Whole body	1.26 (1.01 to 1.58)	**0.04**	1.40 (1.02 to 1.91)	**0.03**

^a^The multivariate logistic regression model included age, BMI: gender, education, income/year, hyperlipidemia, hypertension, diabetes, smoking, passive smoking, drinking, and the phase angle at a frequency of 50 kHz. *P* values of < 0.05 were considered statistically significant.

**Table 4:** The associations between CAP and phase angles at a frequency of 50 kHz.

Phase angles	*β* (95% CI)		OR (95% CI)	
	Univariate analysis	Multivariate analyses^a^	Univariate analysis	Multivariate analyses^b^
Right arm	6.11 (1.17 to 11.06)^*∗*^	1.40 (−4.42 to 7.23)	1.38 (1.13 to 1.7)^*∗∗*^	1.09 (0.83 to 1.43)
Left arm	7.24 (2.32 to 12.16)^*∗∗*^	2.09 (−3.67 to 7.85)	1.47 (1.20 to 1.81)^*∗∗∗*^	1.21 (0.92 to 1.58)
Trunk	5.20 (2.45 to 7.94)^*∗∗∗*^	2.93 (-0.09 to 5.95)	1.27 (1.13 to 1.44)^*∗∗∗*^	1.13 (0.98 to 1.31)
Right leg	8.72 (4.51 to 12.93)^*∗∗∗*^	9.69 (4.79 to 14.59)^*∗∗∗*^	1.46 (1.22 to 1.74)^*∗∗∗*^	1.45 (1.15 to 1.82)^*∗∗*^
Left leg	8.86 (4.53 to 13.19)^*∗∗∗*^	9.40 (4.33 to 14.46)^*∗∗∗*^	1.47 (1.23 to 1.76)^*∗∗∗*^	1.43 (1.13 to 1.81)^*∗∗*^
Whole body	9.27 (4.37 to 14.17)^*∗∗∗*^	7.20 (1.32 to 13.09)^*∗*^	1.54 (1.25 to 1.89)^*∗∗∗*^	1.34 (1.02 to 1.76)^*∗*^

^a,b^ Adjusted for age, BMI, gender, education, income/year, hyperlipidemia, hypertension, diabetes, smoking, passive smoking, and drinking.^*∗*^, *P* < 0.05;^*∗∗*^,*P* < 0.01;^*∗∗∗*^, *P* < 0.001.

**Table 5:** Multivariate logistic regression analyses of phase angles at a frequency of 50 kHz with NAFLD and abnormal CAP, stratified analyses by BMI.

Phase angles	NAFLD: OR (95% CI)^a^		Abnormal CAP: OR (95% CI)^b^	
	BMI <30	BMI ≥30	BMI <30	BMI ≥30
Right arm	1.23 (0.89 to 1.68)	0.57 (0.19 to 1.75)	1.12 (0.83 to 1.52)	1.01 (0.55 to 1.85)
Left arm	1.33 (0.97 to 1.82)	0.68 (0.24 to 1.98)	1.29 (0.96 to 1.75)	1.07 (0.59 to 1.94)
Trunk	1.18 (1.01 to 1.39)	1.04 (0.55 to 1.97)	1.22 (1.04 to 1.43)^*∗*^	1.02 (0.71 to 1.46)
Right leg	1.70 (1.30 to 2.22)^*∗∗∗*^	0.66 (0.27 to 1.60)	1.58 (1.22 to 2.05)^*∗∗∗*^	1.39 (0.82 to 2.35)
Left leg	1.53 (1.17 to 2.00)^*∗∗*^	0.63 (0.25 to 1.61)	1.49 (1.15 to 1.93)^*∗∗*^	1.61 (0.92 to 2.79)
Whole body	1.65 (1.20 to 2.28)^*∗∗*^	0.58 (0.19 to 1.76)	1.47 (1.08 to 2.00)^*∗*^	1.20 (0.65 to 2.24)

^a,b^ Adjusted for age, BMI, gender, education, income/year, hyperlipidemia, hypertension, diabetes, smoking, passive smoking, and drinking.^*∗*^, *P* < 0.05; ^*∗∗*^, *P* < 0.01;^*∗∗∗*^, *P* < 0.001.

## Data Availability

Data are available upon request to the corresponding author.

## Supplementary Materials

Supplementary Materials Table S1: correlations of phase angles among different body parts.
