# Supplementary material for: Associations between Phase Angle Values Obtained by Bioelectrical Impedance Analysis and Nonalcoholic Fatty Liver Disease in an Overweight Population
Source: Can J Gastroenterol Hepatol. 2020 Aug 4;2020:8888405. doi: 10.1155/2020/8888405 (PMC7426783; doi:10.1155/2020/8888405)
Supplement: Supplementary Materials — Table S1: correlations of phase angles among different body parts. [file 8888405.f1.docx]

**Table S1.** Correlations of phase angles among different body parts

| **Phase angles** | Right arm | Left arm | Trunk | Right leg | Left leg | Whole Body |
| --- | --- | --- | --- | --- | --- | --- |
| Right arm | — | 0.94 | 0.61 | 0.68 | 0.69 | 0.92 |
| Left arm | 0.94 | — | 0.59 | 0.64 | 0.65 | 0.87 |
| Trunk | 0.61 | 0.59 | — | 0.56 | 0.56 | 0.68 |
| Right leg | 0.68 | 0.64 | 0.56 | — | 0.93 | 0.90 |
| Left leg | 0.69 | 0.65 | 0.56 | 0.93 | — | 0.86 |
| Whole Body | 0.92 | 0.87 | 0.68 | 0.90 | 0.86 | — |

All *P-*values < 0.01 from Pearson’s correlation test.
